# Notes of the Tyler Meeting T. S. M. A.

**Published:** 1892-05

**Authors:** 


### NOTES OF THE TYLER MEETING.

It was the intention of the the manufacturers to have one of the unique Nedofik sofas there, but Dr. Keoun, the agent, failed to connect.

The irrepressible and popular Mr. Cox was there, as usual, with a full stock of sweet smiles and Mellin's Food, both of which he dipensed with courteous grace.

Messrs. Park Davis & Co. were, as usual, represented by their courteous agents and a full display. This house is a universal favorite, and everybody knows and uses their products.

The sore-heads were conspiculously absent, not one of them was present except "that-man-Burch." He kept quiet, however, missing the "sick-im" of his "kingly beast."

Amongst the numerous displays of pharmaceuticals that of Sharp & Dohme, under the charge of those courteous gentlemen, Dr. Winchester and the "Duke" (Mr. Wellington), was conspicious for elegance of preparation and taste in arrangement. Especially worthy of trial is "Ergotol," a standard strength, uniform fluid preparation of ergot, far more reliable and convenient than ergotin, or the fluid extract of ergot.

The JOURNAL begs here to acknowledge the cordial reception and delightful hospitality extended its editor by Dr. W. J. Goodman, a retired physician of Tyler, and his amiable wife and charming young daughter, at their lovely suburban home. The three days stay with this typical Southern family—and their unceasing attentions, which were simply delightful, will ever constitute a green spot in our memory. It alone more than compensates us for the time and trouble of the trip.

The Section officers elected at this meeting, it is to be hoped, will appreciate the honor conferred upon them and show their appreciation by working to get up a better lot and more papers than their predecessors did for the Tyler meeting. We believe, only two chairmen were present, and the papers in all the Sections, except those of Surgery and Practice, were few and not of the highest order of merit. The report by Dr. Knox, chairman of Section on Dermatology, may, without prejudice to other papers, we hope, be mentioned as one exception at least. That of Dr. Cunningham, chairman of Section on State Medicine—as another. We will not say, however, that these were the only exceptions.

Dr. James Kennedy, of San Antonio, against whom the West Texas Medical Association brought charges of violation of the code of medical ethics—in that it was announced in a newspaper that he had performed an operation—was "exonerated." The father of the patient had inserted the notice. The West Texas Medical Association had expelled Dr. Kennedy, and on evidence presented to the Judicial Council, that tribunal decided the act of expulsion was illegal, and referred the matter back to the society. Dr. Kennedy had gone to the courts with his case, and applied for mandamus to reinstate him. We hope the matter will be satisfactorily arranged. Dr. Kennedy is a useful working member of the State Association.

The Address of the President, although an excellent one, was to us somewhat of a disappointment;—the choice of a subject we regarded as unfortunate,—"the Progress of Medicine." Although handled as well as could well be, our expectations had been so high that we were a little disappointed. Dr. Wilkes is so well known as an original and witty speaker that something in that line, something out of the ordinary, was expected. The "Progress of Medicine" is so hackneyed that it is really tiresome, especially to one who reads the journals and keeps pace with the progress; but, to the laity, and especially to ladies, it must have sounded like a fairy tale, so wonderful indeed has the progress been. Wonder how many, except doctors, can comprehend the idea of a combat between blood-cells and invading pathogenic bacteria?—Truth is stranger than fiction.

We take pleasure in referring to the display of surgical instruments made at Tyler, Texas, by the Fort Worth Pharmacy Co., of Fort Worth. Perhaps it was our own feeling of State professional pride that made this display look more attractive to us than any other. But, beyond question, the exhibit of this firm was very attractive, and not only a subject of comment, but one of congratulation by the members of the convention that we had within our State such a stock of surgical goods, so readily at the command of the profession by telephone, telegraph or letter. We carefully examined the prices of their goods, and found them equally as low as asked by any of the Eastern surgical instrument firms. It is clearly to the interest of the profession of Texas to patronize and support the Fort Worth Pharmacy Co., for they have already gone quite near to the front. If you have not their illustrated catalogue write for it.

Dr. J. D. Osborn, the President-elect, is a general favorite with the profession. He is a handsome man with courtly address, and will doubtless reflect credit on the Association as its presiding officer. His election gives general satisfaction, and indicates a new order of things. Representing as he does the later generation of practitioners—the element of progress—we hope to see much improvement in interest in our meetings.

If the honors may be regarded in any sense as rewards, the presidency could not have been more worthily bestowed; for Dr. Osborn has been regular in his attendance and faithful in the discharge of every duty imposed upon him—whether as judicial council-man, committee-man, or chairman or secretary of any Section, and he has filled all these positions. The Association is to be congratulated upon so judicious a choice.

Dr. Osborn is, and for several years has been, State Medical Examiner of the Order of Knights of Honor, and has figured some in local politics, but has never held any political office so far as we know,—preferably devoting himself to his profession.

Dr. T. J. Bell, of Tyler, the First Vice-President-elect, is another happy choice. As a working member Dr. Bell has ever been distinguished. He is also a young man, comparatively, and will do honor to the position. His election is a graceful acknowledgment of his efficient services as President of the Judicial Council at Waco—a most trying position, which he filled with credit to himself and satisfaction to the Association.

The Second and Third Vice-Presidents, Drs. F. M. Pitts, of McCalm, and T. J. Bennett, of Austin, the well-known editor of the *Texas Sanitarian*, are prominent and popular members and have each contributed much in the past to the interest and profit of the Association

